# Buffalo Medical and Surgical Association

**Published:** 1885-03

**Authors:** Clayton M. Daniels

**Affiliations:** Secretary pro tem.


### Society Reports.

### Buffalo Medical and Surgical Association.

*Regular Monthly Meeting, Jan. 6, 1885.*

President, Dr. F. W. Bartlett, in the Chair.

Present—Drs. Cronyn, Hubbell, Tremaine, F. H. Potter, W. W. Potter, Dorland, Crego, Fowler, Brecht, Strong, Stockton, Berkes, Thornton, Hopkins, Van Peyma, Daniels, Coakley and Pryor.

Minutes of last meeting were read and approved.

Essay by Dr. C. G. Stockton, entitled, "Some Untoward Effects of Opium."

*Discussion*—Dr. Strong considered that the case described was very instructive. He thought that during the first stage the patient would bear more than when the reaction had occurred, as there would then be another state of the system.

Referring to suppositories, he thought we did not know what proportion was absorbed. There had been, in the case stated, doubtless,a large accumulation of opium in the bowel. Considered opium a wonderful drug. He always felt elated when he saw its wonderful action. He would as soon think of going into battle with one arm tied behind him, as to practice medicine without opium. He feared that mistakes had been made in estimating doses, and that it had been given too largely, and believed we should only use it when absolute weight is given, and be especially careful in its hypodermic administration. Speaking of the tolerance of the drug, he referred to an astonishing case, of a child of eight months, who had diarrhœa. Paregoric was minutely used at first, and the opiate was gradually increased until, at the end of two weeks, it was receiving half drachm doses of laudanum.

Dr. Dorland expressed his enjoyment at listening to the paper, as it interested all practitioners. His especial choice of opiates was the "Dover's powder." He had been greatly surprised, sometimes, at the effect of some other forms. He advocated the "Syrup of Dover's," for children. Had seen some alarming effects from paregoric. He thought there might be something in the combination of "Dover's powder" (the ipecac) that modified the effects of the opium.

Dr. W. W. Potter came to be instructed, and was not disappointed in his expectations. He had heard of the case referred to, in the paper. Considering the large doses given, he said no man knew the effects of drugs better than the late Dr. White, and that as the case was under his control and direction, his judgment could hardly be questioned. Dr. Potter thought it wise to administer atropia in connection with opium. The popular idea was that opium, hypodermically, was dangerous, but he considered it safest where the dose was properly graded. Referring to idiosyncrasies, he thought that giving opium in highly neurotic cases was not good practice. But sometimes, where pain was great, that it would then be well borne.

Dr. Hubbell said Dr. Stockton had referred to one case where opium had produced very great excitement. He had one case himself, as follows: Boy thirteen or fourteen years old, previously had inflammatory rheumatism with some endocarditis and valvular lesion. After a time pain and angina appeared; at one time a paroxysm was very severe, and he administered morphia in one-sixth grain doses every two hours for three doses. The first produced excitement, the second increased it, and after the third the patient became fully delirious, scarcely knowing who were around him. He then gave chloral and bromide of potash, five grains each, in solution, every half hour. At the end of one and one-half hours, the patient became quiet and went to sleep; at the end of the third hour, pain subsided, and he made a good recovery from that attack. He thought this corroborated the case of Dr. Stockton regarding "Some Untoward Effects of Opium."

Dr. Cronyn remarked that the effects of opium were known; accidents were constantly happening which were not so fortunate as the cases of Drs. Stockton and Hubbell; opium was not the same to all. The idiosyncrasies were, with all range of drugs, the same. Opium's well-known action is stimulant, anodyne, soporific and narcotic, but may occur in opposite degree with different individuals.

He had known those who could take large doses, and again others who could not take it at all. Before prescribing it he always tried to find out if the patient had ever taken it before.

He believed in the curative effects of the crude opium, and recommended quinine in combination with it, believing it to be a valuable adjunct; advised others to try it. Where opium has been given to those who illy bore it he thought the utmost caution should be used in following it with chloral, as ten grains of chloral with one-sixth grain of morphia is more potent than thirty grains of chloral alone. Regarding the absorption of opium by the rectum, he said that many agents were as active there as in the stomach, chloral being one. He did not think the hypodermic method more safe than by the stomach, for the reason that an emetic could not be used, or the dose in any way reached or neutralized. He had combined morphia with atropia, giving small doses of morphia frequently repeated, beginning with one-twelfth grain and repeating every fifteen to seventeen minutes.

He supposed that opium was among those medicines that were cumulative in character. He had seen a case of opium excitement controlled by placing a very brilliant metal before the eyes of the patient Opium had been used to quiet nervous irritability of a maniacal character. Believed that in opium we had a medicine of great value, but that we could not be too cautious in its administration.

Dr. Tremaine had once heard a keen observer say that when a doctor had solved the problem of the proper administration of opium he had learned how to practice medicine.

Dr. Van Peyma believed that much depended upon the form of opium used, and that some were more harmful than others; considered its combination with other drugs advisable. He had tried, in one case, the large bright object referred to by Dr. Cronyn, but without success.

Dr. Crego had observed the action of morphia and hyoscyamin. He was once called to see a young lady that had delusions; as she was taking medicine that he believed to contain opium, he ordered it discontinued, and in thirty-six hours the delusions had disappeared. Had seen similar conditions after very small doses of the drug, Among the insane, where he had opportunities for observation, he had noted that it seemed to increase the excitement. In melancholia, if given largely, it quiets; in small doses it excites. Recommended that it be combined with bromide of potass. He had seen no unpleasant effects from opium and chloral combined, but had been cautious. Would give the pot. brom. with it. He had noted that morphia, in small doses, often repeated, did not seem to agree so well as large doses longer apart. Opium was eliminated from the system much more slowly than the bromide or chloral. In a paper which he read some time since he had tried to point out that where morphia would not answer hyoscyamin often would, as he had seen it practically demonstrated. He considered the amorphous preparation of hyoscyamin the best. Speaking of the excessive quantities of opium that could be taken, he had a recent case where the patient took sixty grains daily. In such cases the pulse becomes very slow and weak; he had seen it go to thirty-four per minute; and on the skin the symptoms were an intolerable itching and there were, frequently, discolorations which were peculiarly characteristic.

Dr. Coakley said that during the war opium, quinine, calomel and whiskey constituted the surgeon's materia medica. The soldiers would tolerate opium largely. He attached great value to it, but of late years had at times regretted its use. Thought it required the greatest discrimination to decide when it ought to be used. In his own practice he had a case of excessive alcoholism, and patient asked for an opiate. He gave him, as he supposed, about one-third, and certainly did not exceed one-half grain of morphia. In about three hours patient attempted to go across the floor and dropped dead from apoplexy. He could not say the morphia was the cause but it was an unpleasant occurrence and made him cautious. He had seen a case where the doctor gave a woman who was eight months pregnant, one-sixth to one-fourth grain of morphia hypodermically; she appeared to have opium poisoning and died very soon. He now used the drug preferably by the bowel in suppositories, combined with belladonna, but limited its use as much as possible, preferring to substitute bromides, chloral, etc.

Dr. Hopkins had seen some of the untoward effects of opium, and especially referred to two cases of his own where a peculiar condition followed immediately upon a hypodermic injection of morphia, a red glow coming upon every portion of the exposed skin, pulse very greatly accelerated, going up to two hundred and faster than could be counted, the blood seeming to rush to upper portion of body in great quantity, all this occurring before it was possible for the solution to be absorbed, and almost before the needle had been withdrawn. All the alarming symptoms passed away in about five minutes, doing absolutely no harm. He believed the action was upon the nervous system, on account of the symptoms appearing before absorption could occur. He wished to dissent from the present form of discussion, as it was not confined to the subject.

Dr. Bartlett, in referring to Dr. Hopkins' remark, said that we were likely to drift into personalities, which it was hard to avoid. One of the untoward effects of opium was the habit of its use. It was usually given first for temporary relief, and it grew until the victim was unable to discontinue its use. He thought the habit more frequently followed hypodermic medication. Referred to a case where great deception was practiced in order to obtain it in this manner, and where it was estimated that the man received more than fourteen hundred injections within two years, some being more than two grains each. He was against giving opiates in any form in childhood where it could possibly be avoided, as he believed the habit and demand for the drug could be then formed. Had seen a case where a child of six months had been given a teaspoonful of laudanum every night for some time. He had also attended a family where the man took sixty grains of morphia daily. As to hypodermic use of opium, the number of deaths that have occurred in this city from it should cause us to use the greatest caution. One-eighth of a grain had caused death of a case in his own practice. In nervous people he found that svapnia was well borne. Some had recommended large doses of opium in eclampsia, but he did not believe in it.

*Prevailing Diseases*—Diphtheria, scarlatina, rheumatism, influenza, pharyngitis, and Dr. Stockton had seen some cholera morbus.

A letter of resignation from Dr. Peterson, the Secretary of the association, which had been received by the President, was read, and, upon motion, his resignation was accepted.

Balloting for a Secretary being in order, Dr. F. H. Potter was elected Secretary to fill the vacancy.

Adjourned.

CLAYTON M. DANIELS, *Secretary pro tem.*

